# Study of Flow Rate Measurements Derived from Temperature Profiles of an Emulated Well by a Laboratory Prototype

**DOI:** 10.3390/s19071498

**Published:** 2019-03-28

**Authors:** Werbet L. A. Silva, Verivan S. Lima, Diego A. M. Fonseca, Andrés O. Salazar, Carla W. S. P. Maitelli, German A. Echaiz E.

**Affiliations:** 1 Laboratório de Avaliação de Medição em Petróleo, Universidade Federal do Rio Grande do Norte, Natal 59072970, Brazil; verivan@petrobras.com.br (V.S.L.); diegomoura@dca.ufrn.br (D.A.M.F.); andres@dca.ufrn.br (A.O.S.); 2 Laboratório de Automação em Petróleo, Departamento de Engenharia de Petróleo, Universidade Federal do Rio Grande do Norte, Natal 59072970, Brazil; carlamaitelli@gmail.com; 3 Departamento de Automatización y Control, Escuela de Ingeniería Electrónica, Universidad Nacional de San Agustin de Arequipa, Arequipa 04002, Peru; gechaiz@unsa.edu.pe

**Keywords:** injection profile, wellbore, prototype well, flow measurement

## Abstract

The objective of this work is the study of the procedure for flow rate measurement derived from temperature profiles. This method is deemed appropriate because of the inability to mount conventional flow meters in multiple-zone oil wells. In this work, a reduced-scale prototype well with horizontal geometry was developed to study and validate a method of flow profiling by temperature measurements in the well column based on a heat transfer mathematical model studied by Ramey in 1962. Flow sensors were installed at key points to provide validation data for the flow measurements derived from the temperature. The plant was automated and all the tests were managed from a workstation. It was possible to test different situations to provide a variability of evaluation scenarios. The initial experiments used injected fluid flow rates of 15 and 18 L/min in the well inlet. The results of the calculated flow values in different work conditions were compared with a relatively low error reference meter.

## 1. Introduction

Waterflooding has become a common technique since its potential to increase oil recovery was recognized. It is considered the most successful method for improving oil production in fields [1,2]. Mongollón [3] highlights in his work that more than a half of the amount of oil in the world today is produced through the waterflooding method.

Real-time monitoring of operational parameters in an injection well is important for production optimization and to diagnose potential problems [4]. When an injection well has multiple production zones, the injection profiles are often used to measure the water flow for each zone. It is the most suitable method to detect and fix anomalies. As a result of the well monitoring process by injection profiles, an improvement in oil recovery and overall financial efficiency can be seen in fields that use waterflooding recovery.

Continuous spinner flowmeters are used to determine the rates of fluid in production and injection wells. Measurement with a spinner is a process that has a high cost and is difficult to execute because it requires a tool to be located at the measurement point in the well column. Moreover, this method does not offer data in a suitable frequency for adequate well operation monitoring whilst also affecting the operation [5]. Because of the growing complexities of well trajectories, running conventional production monitoring tools on appropriate locations has become difficult and costly [6].

In recent years, some models of heat transmission within oil wells have been applied to generate thermal profiles (direct models) [7,8] and to describe the injected flow rates as a function of the fluid-temperature profile (reverse problem) [9]. In addition, in recent years fiber optic distributed temperature sensing (DTS) technology has been used for monitoring injection profiles in wellbores [10,11,12]. This technology has improved the flow rate monitoring process, because DTS, among other benefits [13], provides permanent injection monitoring without production intervention, and can acquire temperature data along the entire extension of the wellbore simultaneously [14]. Therefore, developing a kind of software sensor (as the ones described in References [15,16]) represents an attractive approach for the measurement of flow rates from temperature data.

This work is part of a project that aims to develop a model-based software sensor focusing on the flow measurement process from temperature logs in multiple-zone wells. The first step, realized by Reges [17], applied the model proposed by Ramey [18] to perform zonal flow measurements from fluid temperatures and compared his results with flow rate data from a multiple-zone well collected by a spinner flowmeter. In the second step, discussed in this work, a horizontal, reduced-scale prototype well was designed and built at Laboratório de Avaliação de Medição em Petróleo (LAMP) in Universidade Federal do Rio Grande do Norte (UFRN) to validate the model in different operational conditions. Using conventional instruments, the plant was able to generate test standards with reproducibility to evaluate the quality of the model studied and apply the scientific process to the software sensor improvement.

The contributions of this work for the development of a soft sensor to measure the flow rate in multiple-zone wells are as follows: obtaining an emulated well where it is possible to perform experimental runs in controlled conditions that validate and improve a model that relates the temperature along the wellbore to the fluid zonal allocation. A tool, such as an emulated well, is useful since in real wells it is not possible to carry out experimental validation tests.

## 2. Theoretical Foundation

This section discusses the importance of flow profiles for production optimization in multiple-zone water wells. It also describes the differential equation developed by Ramey [18] that is used in this work as a tool for flow rate measurement in injection wells from fluid-temperature profiles.

### 2.1. Injection Profiles

The wellbores in the oil industry are classified as “simple”, when all the production is concentrated in a single region of the reservoir, and “multiple” in the cases where there are different production zones along the well column. In the second case, the wells are known as multiple-zone wells. In his work, Reges [17] explains the main components used in a water-injection well, the main characteristics of a multiple-zone well, and how the injected water flows to the injection depth intervals.

The continuous monitoring of flow in water-injection wells provides the following: injection rate optimization, leak detection and control, cross-flow detection, the contribution of each zone in the overall production, and the detection of the water breakthrough in the producer well [4]. Specifically, when a well produces across multiple zones, the knowledge about each zone’s contribution in the total injection flow is important for the production management [19]. The presence of natural or induced leaks, or high permeability zones causes an early eruption of water in the producer wells, reducing the sweep efficiency and the well production.

Although important for the production management in wells working with water-injection, there is no local flow measurement methods for the real-time acquisition of flow in the injection points [20]. Usually, a piece of equipment called a production logging tool (PLT) is inserted along the wellbore for the acquisition of the well variables (flow and temperature, for example). The acquired data are analyzed and the injectivity profiles are plotted. It is a high-cost process that does not offer data in at a suitable frequency for the management of the reservoir. In fields where there are multiple-injection wells, the period to obtain all the necessary profiles using a PLT can take days or weeks.

A solution to the flow measurement problem in wells is studied in References [21,22], which worked with the qualitative estimation of injected or produced flow in multi-zoned wells from the thermal profile. From the study of Ramey [18], which proposed a solution to the problem of heat transfer in wells with cold or hot fluid injection, Reges [17] showed the possibility to acquire the injectivity profile in multi-zoned wells from temperature profiles.

Nowadays, the use of DTS is possible to trace temperature profiles in production and injection wells. With this technique, the temperature profiles can be monitored at surface in real time. This approach reduces the need for production logs, prevents deferred production losses, decreases well interventions, and reduces operating costs [23]. Thus, solutions such as those proposed by Reges [17] applied along with DTS systems bring an improvement to the well flow rate monitoring, since it provides real-time and continuous measurement without the use of a downhole sensor and with little to no impact on the well operation [24].

### 2.2. Ramey Model

In his work, Ramey described the temperature of the fluid, tubing, and casing as a function of time and depth. The fluid temperature is a result of the transfer of heat between the formation temperature and the fluid. This phenomena happens in all production operations.

When considering the injection of a non-compressible fluid with known values of temperature and flow in a wellbore, an approximate relationship, obtained from the energy balance applied on the fluid flow in a injection-well, can be given by the follow equation:

(1) $$\frac{dT_{f}\left(z,\dot{m},t\right)}{dz}+\frac{T_{f}\left(z,\dot{m},t\right)-T_{g}\left(z\right)}{A(z,\dot{m},t)}=0$$

where *t* is the time, *z* the depth, $T_{f}\left(z,\dot{m},t\right)$ the average temperature of the fluid, $T_{g}\left(z\right)$ is the formation temperature, and *A* the relaxation coefficient, which is equal to

(2) $$A=\frac{\dot{m}c_{p}\left[k_{f}+U\left(z,\dot{m}\right)rf\left(t\right)\right]}{2\pi rk_{f}U\left(z,\dot{m}\right)}$$

with $\dot{m}$ corresponding to the injected flux, $c_{p}$ to the specific heat of the injection fluid, $k_{f}$ to the thermal conductivity of the earth, and *r* to the inner radius of the tubing. The terms $U(z,\dot{m})$ e f(t) (described by Ramey [18]) are the overall heat-transfer coefficient and transient heat-conduction function, respectively.

## 3. Proposed Model of Prototype Well

This section presents how the prototype-well structure was realized. Furthermore, it shows the qualitative relationship between the flow rate distribution and temperature profile along the wellbore. Through heat-transfer coefficients analyses of the walls of a typical well, a simplified structure was defined for the prototype well. Lastly, the constructed prototype is showcased.

### 3.1. Reference Model

**Figure 1:**
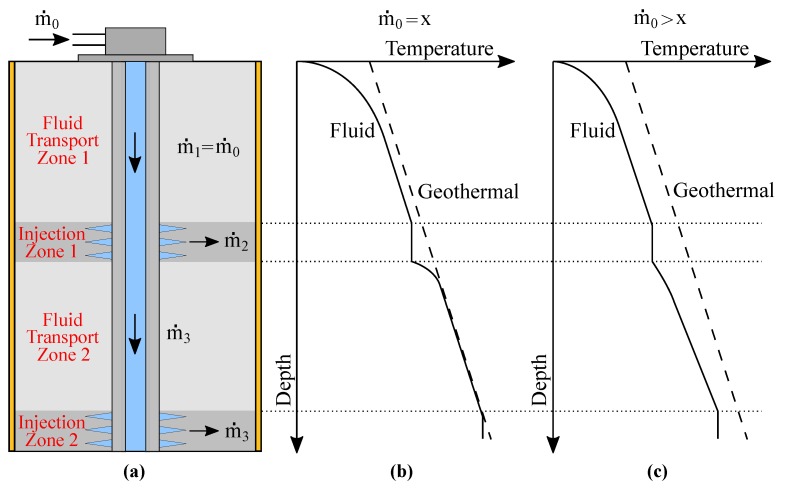
Flow and temperature along a multiple-zone water-injection well used as reference for the completion structure. (**a**) Longitudinal section and the flow distribution in the injection intervals. (**b**) Hypothetical fluid profile for ${\dot{m}}_{0}=x$ as the consequence of the influence of the hypothetical geologic-formation profile. (**c**) Hypothetical fluid profile for ${\dot{m}}_{0}>x$.

Consider the injector well illustrated in Figure 1a.

The well is composed of two injection intervals and two fluid transport zones. The transport zones have the function of directing the fluid to the injection intervals, where it meets the oil reservoir. In Figure 1a, the heat source that creates and maintains the geothermic profile in the well is also represented.

Figure 1b shows a hypothetical flow distribution where ${\dot{m}}_{0}$ is the total flow measured by a traditional flowmeter on the well-head. The flow rate of the first transport zone is the same as the flow rate of the water being injected into the well (${\dot{m}}_{0}={\dot{m}}_{1}$). In order to determine the remaining values (${\dot{m}}_{2}$ and ${\dot{m}}_{3}$), data on flow behavior from a transport or an injection zone are needed. If we suppose the flow rate from the second transport zone is equal to ${\dot{m}}_{3}$, the injected flow in injection zone 1 is determined by ${\dot{m}}_{2}={\dot{m}}_{3}-{\dot{m}}_{1}$. Then, the flow in the second injection zone is the same as ${\dot{m}}_{3}$.

As shown in Figure 1b,c and observed in the oil-fields, in a thermal steady state operation, each flow rate into a transport zone generates a correspondent thermal profile in the fluid, in a one-to-one parity. There is a mathematical relationship between the thermal profile along the well and the flow distribution. Therefore, knowing the thermal profile function, the relationship can be used to derive the correspondent flow distribution [21,25]. From Figure 1, the flow rate ${\dot{m}}_{0}=x$ generates a thermal profile in the injected fluid closer to the geothermal profile when compared to the flow ${\dot{m}}_{0}>x$.

### 3.2. Physical Structure of the Prototype Well

The prototype-well structure was determined from the analysis of the heat-transfer coefficients of a typical well wall. The heat-flux rate through the wall layers in an injection well is proportional to the temperature difference between the injected fluid and the formation, as well as to the perpendicular area to the heat-flux propagation. The overall heat-transfer coefficient considers the net resistance to heat flow offered by the fluid inside the tubing, the tubing wall, fluids or solids in the annulus, the casing wall, and the cement. The heat-flux tax between the fluid and the formation is given by

(3) $$\dot{Q}\left(z\right)=2\pi r_{1}U\left(z\right)\Delta z\left[T_{fluid}\left(z\right)-T_{form}\left(z\right)\right]$$

where $2\pi r_{1}\Delta z$ corresponds to the inner area of tubing in the Δz increment and $T_{form}\left(z\right)$ is the temperature of the completion–formation interface. It is possible to adopt the thermal gradient along an axis parallel to the fluid flow as insignificant when it is compared to the perpendicular thermal gradient [18,26]. At the same boundary conditions as a real well and in a thermal steady state operation, Reference [26] determined that the overall heat-transfer coefficient is given by

(4) $$\frac{1}{U}=\frac{1}{h_{fluid}}+\frac{r_{1}ln\left(\frac{r_{5}}{r_{1}}\right)}{k_{e}}.$$

**Figure 2:**
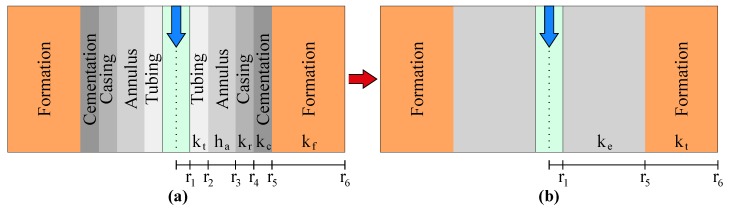
Equivalency between a well commonly used in the oil industry and a simplified structure of a well based on the wall layers conductivity analysis. (**a**) Profile of a typical structure found in the oil industry composed by: cementation, casing, annulus and tubing. The parameters $k_{t}$, $h_{a}$, $k_{r}$ and $k_{c}$ are the respective heat-transfer mechanism coefficients and $r_{1}$, $r_{2}$, $r_{3}$, $r_{4}$ and $r_{5}$ the respective radiuses; (**b**) Simplified structure where all the coefficients are represented by a equivalent parameter $k_{e}$.

The $k_{e}$ value represents a conductivity equivalent to all heat-transfer mechanisms from the internal surface of the tubing to the external surface of the cementation. The $h_{fluid}$ is the convection coefficient of the fluid. This way, it is possible to adopt a simplified completion representation for the well prototype, composed only for the tubing, with no representative losses. This equivalency is shown in Figure 2.

**Figure 3:**
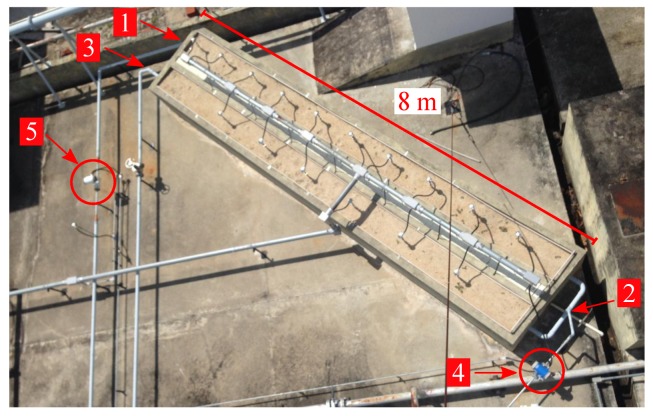
Top view of the prototype well once built. **1**—well-head; **2**—injection zone 1; **3**—injection zone 2; **4**—flow sensor; **5**—flow sensor.

The prototype was built on a horizontal plane, for ease of access to the measurement points. On the basis of the equivalent conductivity found, the well is a steal tube buried into cubic structures where it is possible to emulate different types of formation, through the use of sand, mud and gravel mixes, for example. A top view of the prototype well can be seen in Figure 3.

The general assumptions for the prototype-build process were:

- Horizontal well completion: ease of access to the measurement points, ability to change the emulated formation around the well;
- Hot water injection;
- PT100 temperature sensors distributed along the column;
- A simplified completion structure based on the conductivity equivalences.

## 4. Methodology

This section shows the methodology used to calculate the flow distribution in the prototype well from temperature measurements. It was developed by the resolution of the Ramey [18] differential equation applied to the prototype.

### Ramey’s Solution Applied to the Prototype

For the resolution of Equation (1), it is necessary to define a function $T_{g}\left(z\right)$ that corresponds to the thermal profile of the formation where the well is located. For this work, it is convenient that the thermal profile be constant with the depth, since the prototype well must be maintained at a fixed temperature during the operation. Then, $T_{g}\left(z\right)=T_{g}\left(0\right)$, where $T_{g}\left(0\right)$ is the initial temperature of the structure. Thus, the relationship of the fluid temperature with the depth and time in a well located in a place where there is no temperature variation of the formation along the well column, by the solution of the Equation (1), is provided by

(5) $$T_{f}\left(z,\dot{m},t\right)=T_{g}\left(0\right)+\left[T_{f}\left(0,\dot{m},t\right)-T_{g}\left(0\right)\right]e^{-\frac{Z}{A(\dot{m},t)}}.$$

The relaxation coefficient is the parameter that permits the use of the methodology, presented by Ramey [18], to calculate the flow distribution in wells. This relationship becomes more evident when Equation (2) is represented in the form:

(6) $$\dot{m}=\frac{2\pi rk_{f}U\left(z,\dot{m}\right)}{c_{p}\left[k_{f}+U\left(z,\dot{m}\right)rf\left(t\right)\right]}A.$$

Equation (5) also relates the thermal profile and the relaxation coefficient. Hence, it is possible to obtain the relaxation coefficient average value ($\bar{A}$) for all fluid transport zones, from the well temperature data. Thus, independent of the flow state, using Equation (6), it is possible to determine the injection flow values ${\dot{m}}_{i}$ from the relaxation coefficient values $A_{i}$ computed in each temperature measurement point along the well column. The well-head flow rate is known and is the reference value ${\dot{m}}_{ref}$. The temperature profile measured in the first fluid transport zone gives an average relaxation coefficient (${\bar{A}}_{ref}$). The flow values in the unknown points are calculated by

(7) $$\dot{m}≅\frac{\bar{A}}{{\bar{A}}_{ref}}{\dot{m}}_{ref}.$$

The manipulation of expression (5) provides the following:

(8) $$ln\left[\frac{T_{fluid}\left(0\right)-T_{g}\left(0\right)}{T_{fluid}\left(z\right)-T_{g}\left(0\right)}\right]=\frac{z}{A}.$$

We defined the left side of Equation (9) as a new variable: the dimensionless temperature ($T_{fluid}^{*}\left(z\right)$). Then,

(9) $$T_{fluid}^{*}\left(z\right)=ln\left[\frac{T_{fluid}\left(0\right)-T_{g}\left(0\right)}{T_{fluid}\left(z\right)-T_{g}\left(0\right)}\right].$$

**Figure 4:**
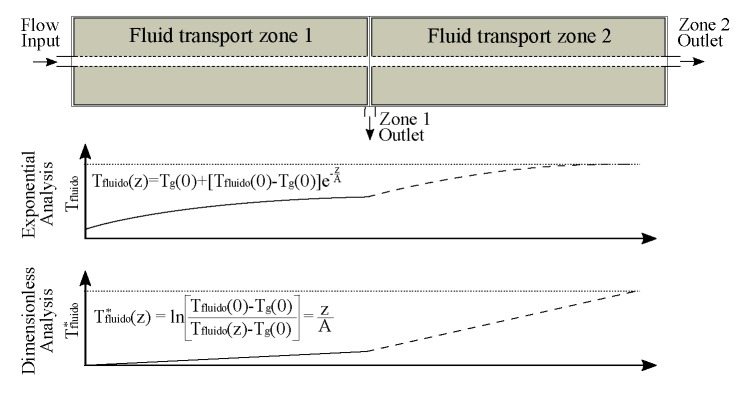
Exponential and dimensionless temperature behavior related to the prototype-well fluid transport zones.

Figure 4 shows a theoretical representation of the prototype. In this approach, the fluid temperatures, measured in the well column, behave as shown in Figure 4 on the “exponential analysis” graphic. In the dimensionless analysis, the thermal profiles have a linear behavior (Figure 4). Thus, the average relaxation coefficient ($\bar{A}$) is obtained from the inverse value of the $T_{fluid}^{*}\left(z\right)$ curve angular coefficient.

Applying the Ramey methodology [18] to compute the injection profile, conforming to the flow state conditions, consists in:

1. Acquiring the temperature profiles with the instruments installed in the well.
2. Applying Equations (5) or (9) to determine the relaxation coefficients for each temperature measurement point.
3. Computing the average relaxation coefficient values.
4. Using Equation (7) to obtain the flow of fluid in each injection point.

## 5. Instrumentation Diagram

**Figure 5:**
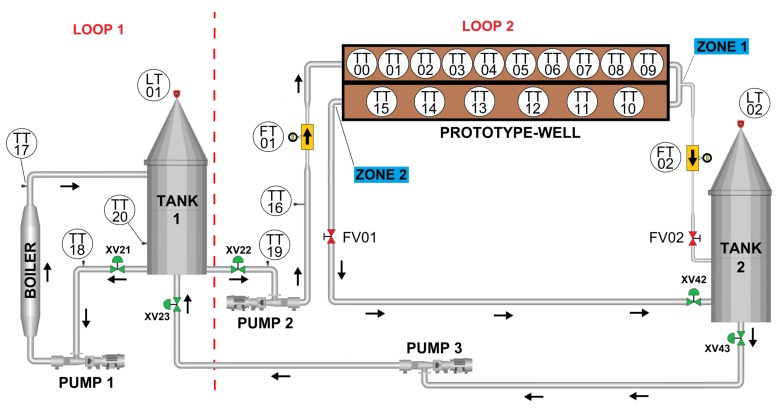
Instrumentation diagram of the experimental plant with an indication of the operation loops.

This section describes the instrumentation diagram (Figure 5) of the plant and the process flow for the measurement tests in the prototype well. Furthermore, the logical and field components installed and their functions to the process flow are detailed.

The system is composed of 21 temperature sensors, two flow sensors, two level sensors, three pumps, seven flow valves, two storage tanks, and a water heater. A programmable logic controller and a dedicated temperature controller serve as the interface between the plant and a supervisory system.

The general operation is divided in two loops: loop 1 heats the water used in the tests; loop 2 transfers the water to the prototype well. The arrows in Figure 5 indicate the direction of the water flow.

### 5.1. Loop 1 Operation

The operation starts by opening valve XV21 (tank 1 outlet) and turning pump 1 on. The water passes through the boiler and returns to tank 1 with a higher temperature. The moment when the set point of the water temperature on tank 1 is established indicates the start of loop 2 operation.

### 5.2. Loop 2 Operation

The flow measurement takes place in loop 2. The hot water comes from loop 1 through valve XV22 and pump 2, then goes to the prototype-well inlet. At this stage of the loop, the water temperature and flow rate are acquired by the TT16 and FT01 sensors.

The prototype-well column is buried in a cubic structure with a mix of sand and mud. This mix simulates the geological formation where the well is to be installed. Along the emulated-well column, there are 16 temperature sensors (from TT00 to TT15) to measure the water temperature variation (temperature profile). The variation results from the heat-transfer from the water to the geological formation.

In Figure 5, the first injection interval (zone 1) divides the well into two transport zones. At this point, there is another flow meter (FT02) and a control valve (FV02). The flow meter registers the flow injection at injection zone 1. The change in the flow passage of FV02 modifies the water flow rate and creates a different operating condition in the well. If the FV02 is totally closed the prototype well works as a “simple” well. Then, the forced change in the water flow creates a different temperature profile along the well column and provides different test conditions.

At the end of the test, all the water is in tank 2. To restart the operation, pump 2 sends the water back to tank 1.

The sensors FT01 and FT02 provide the flow distribution in real time in the prototype-well structure. This information is assumed to be the real values and is compared to the flow measurements obtained from the temperature.

### 5.3. Heater

**Figure 6:**
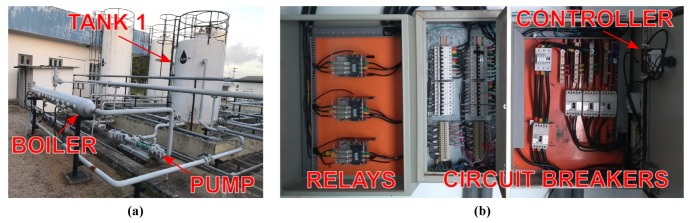
Water heating system. (**a**) Heating plant: boiler, pump, and tank. (**b**) Heating electrical panel.

An electric heater was designed and installed to heat the water used in the tests. Figure 6a shows the real structure of the heating system and Figure 6b shows the power supply circuit that controls the heating operation. There is a closed control loop to maintain the tank 1 temperature at the desirable value. This control loop is composed of: a tank, a boiler, and a pump. The power switches, circuitry breakers, and the automatic temperature controller are installed inside the electrical panel.

The boiler measures 4 meters in length, 12 inches in diameter, and 300 liters in volume. The heater actuators are eight immersion resistors that convert electrical energy to thermal energy. Each resistor demands 9 kW from the power grid and the total power of the heater is 73 kW. The pump has two functions: to transport the fluid from tank 1 through the boiler and mix the water in the tank trying maintain a uniform distribution of temperature in the tank. A temperature sensor acquires the water temperature in tank 1 in real time and sends this information to the dedicated temperature controller. A PID (proportional–integral–derivative) routine calculates the suitable control signal to change the relay operation state. The dedicated controller concentrates all process data and sends the information to other controllers and to the workstation through an internal communication network.

### 5.4. Integrated System

**Figure 7:**
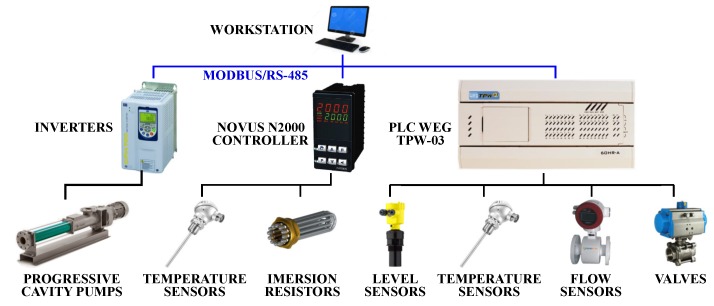
System architecture of the test plant.

The entire system architecture is shown in Figure 7. Three inverters (model WEG CFW-11) control the pumps rotation and power state. The novus N2000 controller controls the actuators of the heat system and monitors tank 1 temperature with a PT100 sensor. A PLC (programmable logic controller) WEG TPW03 collects information as regards tank levels, flow rates and temperatures of the system, and controls the valves. This equipment is important since it provides the interface between the field components and the workstation.

**Figure 8:**
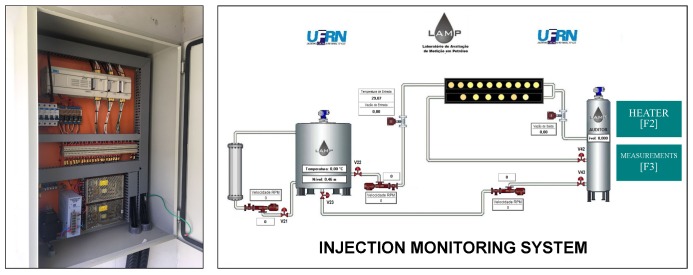
(**a**) Command panel of the PLC WEG TPW03. (**b**) Principal screen of the supervisory system.

All controllers are connected in a serial network that communicates with the workstation. The supervisory was implemented (Figure 8) in a demo version of Elipse SCADA (Elipse Software, Porto Alegre, Brazil).

The application displays a time-series of all the process variables. Furthermore, the software stores the data and controls the operation state of all field components. Figure 8a shows the command panel where the PLC was installed and Figure 8b displays the main screen of the supervisory system.

## 6. Results

The experiments can be divided into two steps: the first one refers to the operational execution of the injection experiment on the test plant. The second one consists of the flow rate estimation along the well by the analysis of the data acquired in the first step. This section describes some results obtained from initial experiments in the plant. For the initial experiments in the new structure, the main objectives were to validate the emulated well and the general system operation, and to identify points of improvement. In this way, a low level of error was not required at that stage. At this point, we expected an indication that the results reflected the theory in some capacity, enabling the progress of the research.

A experimental routine was executed, which yielded temperature and flow data over 3200 s. We divided the data analysis into two types: Type “A”, when all injection fluid was directed to the production interval 2 to obtain a “simple” well completion; and “B”, to obtain a multiple-zone well, i.e., to inject fluid into the two zones.

**Figure 9:**
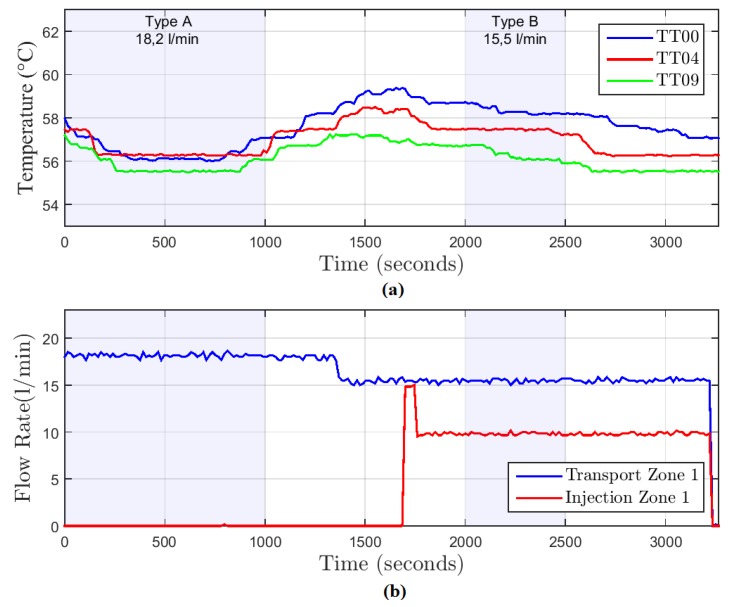
Graphs of temperature and flow rate with time. (**a**) Graph of temperature with time along the transport zone 1. (**b**) Graph of flow rate with time in transport zone 1 and injection zone 1.

In the first step of the data analysis, it is necessary to define the time intervals to be used for flow rate estimation. Figure 9a shows the graph of temperature with time from the sensors TT00, TT04, and TT09. Figure 9b indicates the real flow rate measured by sensors FT01 and FT02.

The graphs in Figure 9 indicate the temperature behavior during each type of test. They help the identification of intervals where there is no significant temperature variation. On this basis, the test windows chosen were:

- Test A (18.2 L/m in the well inlet): samples 0 to 1000.
- Test B (15.5 L/m in the well inlet): samples 2000 to 2500.

As displayed on the process diagram (Figure 5), the flow rate measured by the sensor FT01 corresponds to the flow rate in transport zone 1. While the value given by the FT02 corresponds to the injection zone 1 flow rate. Therefore, the flow rate in transport zone 2 is given by the difference between the flow rates in the sensor FT01 and FT02. For test type B, the flow rate in transport zone 2 was 5.6 L/min.

**Figure 10:**
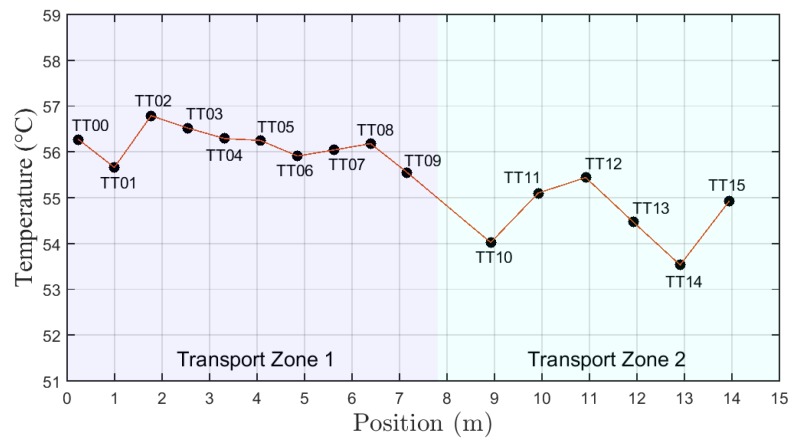
Graph of temperature with position (temperature profile) for test type A.

Once the test windows were defined, the following step was used to plot the temperature profiles. The average value for each temperature sensor in the region defined by the test windows were considered. Figure 10 shows the temperature profile along the well column for test A.

**Figure 11:**
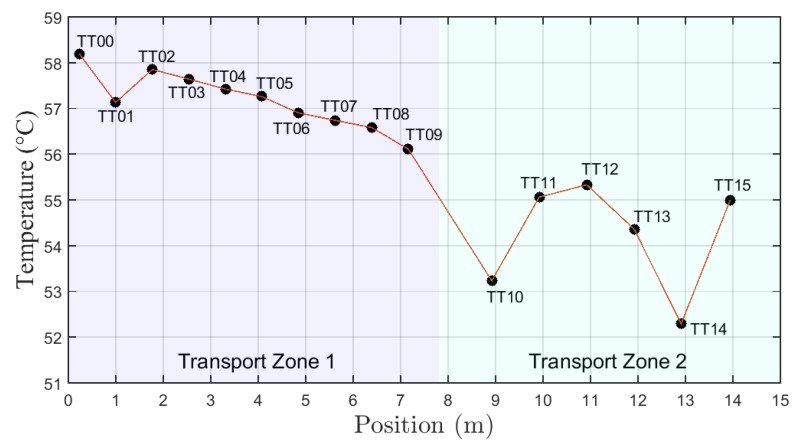
Graph of temperature with position (temperature profile) for test type B.

Figure 11 shows the temperature profile along the well column for test B.

The formation temperature considered was defined by the average value of the temperature of all sensors before the start of the tests and its value was 31.48 C.

It was observed that some temperature sensors presented unexpected behavior, probably because of the incorrect installation of the temperature sensor protection wells, as well as the difference between the distribution and quality of the sand along the prototype well. In addition, the temperature at the prototype border, where the humidity is higher than at the center, contributes to the difference in dynamic behaviors of the sensors and acts to disperse the temperature values. For estimation analysis, the values of TT00, TT9, TT10, and TT15 were not considered.

**Figure 12:**
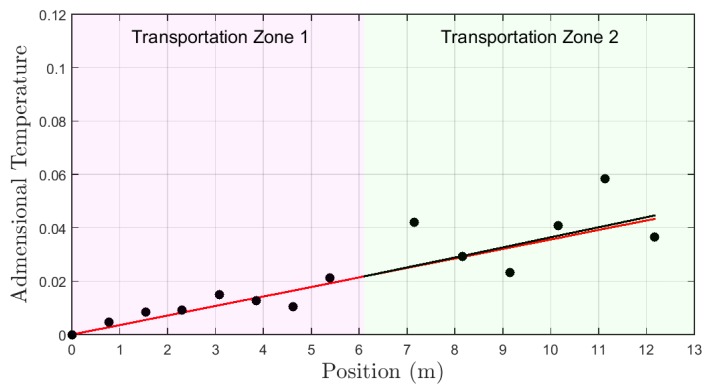
Dimensionless profile—experiment type A.

The dimensionless profile for test type A is shown in Figure 12 represented by black dots. The red curve represents a first order approximation by the dimensionless temperatures for transport zone 1. The black curve represents a first order approximation by the dimensionless temperatures for transport zone 2. In this test, the flow rate in transport zone 1 is equal to transport zone 2. The estimated flow in transport zone 2, by the application of the methodology presented in the Section 4 and the comparison with zone 1 was 17.2 L/min. It represents an error of 5.5% related to the real values measured by the sensor in the well inlet.

**Figure 13:**
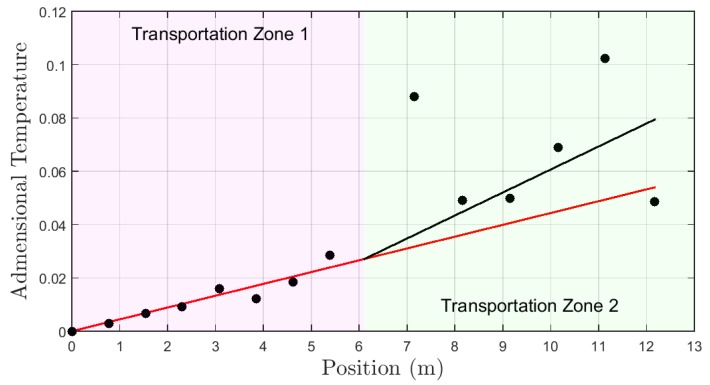
Dimensionless profile—experiment type B.

The dimensionless profile for test B is shown in Figure 13. As for test A, the relationship between the red and black curves indicates the information about the flow rate in the transport zones. The estimated flow rate was 7.9 L/min and the error was 41.1%.

**Figure 14:**
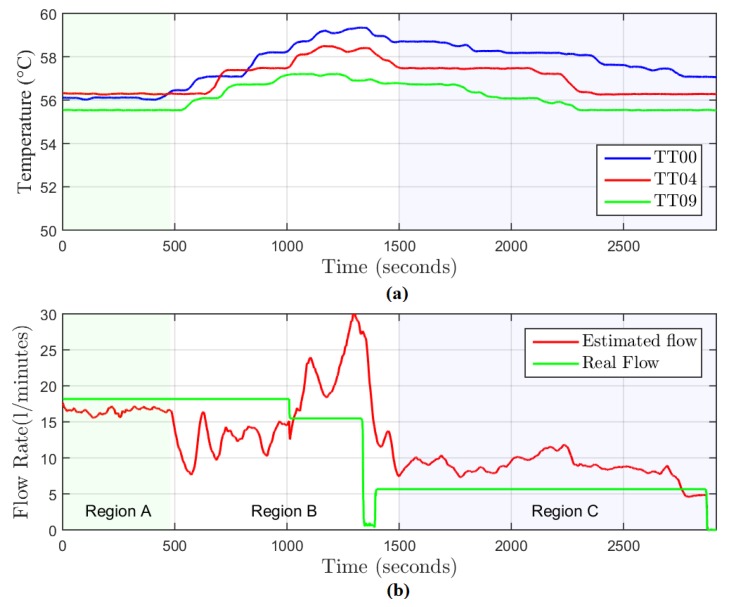
Graphs of temperature and flow with time. (**a**) Graph of temperature in transport zone 1. (**b**) Comparison between the real and the estimated instantaneous flow rates.

The graphs in Figure 14 relate the temperature versus time graph and an estimation of the instantaneous flow rate measurements. We divided the time behavior into three regions (Figure 14b) where the mean error was computed. For region A, it was 9.32%, for region B, it was 53.87%, and for region C, it was 51.42%.

As shown in Figure 14a, it was not possible to maintain the temperatures at a constant value in regions B and C. We observed that the mono-variable heating control system adopted for the initial experiment was not efficient for this application, since it was not able to regulate the temperature of the inlet water to a constant value when the level of tank 1 started to decrease. A constant temperature value is necessary since it is used as the reference to the model. As we see in Figure 14b, in the regions which present greater temperature variations, the estimated flow rates are further from the real values.

In summary, we attribute the errors found to the follow points:

- Incorrect installation of the temperature sensor protection wells.
- The temperature sensors closer of the prototype well behaved unexpectedly. This was probably due to the interaction between the prototype structure and the outside environment being different from that of the internal environment.
- Irregular sand distribution along the prototype well, creating zones with different heat-transfer coefficients.
- Impossibility of maintaining a constant fluid temperature in the well inlet. We observed that the mono-variable temperature control used could not deal with parameter changes (level of tank, for example) during the experience.

## 7. Conclusions

This paper proposed an instrumented prototype well in reduced scale built for the evaluation of the technique developed by Ramey in 1962, applied in the inference of flow information from thermal profiles of water injection wells.

The initial experimental results of flow estimation on the emulated well enabled the acquisition of temperature profiles for different conditions. The obtained results indicated relatively low flow errors in the regions where the temperatures showed a more stable behavior. Despite the levels of error at some points during the operation, we consider that the initial tests were satisfactory since the results of the data analyses enabled the qualitative relationship between the flow behavior and the temperature profile to be studied, in accordance with the methodology proposed by Ramey.

Furthermore, the experiments provided practical knowledge about the system built. Thus, it was possible to identify key points that need structural and operational improvements. In future works, we intend to perform structural adjustments to reduce the level of uncertainty of the flow measurements.
